# Complete Genome Sequence of *Actinobaculum schaalii* Strain CCUG 27420

**DOI:** 10.1128/genomeA.00880-14

**Published:** 2014-09-04

**Authors:** Rikke Kristiansen, Morten S. Dueholm, Steffen Bank, Per Halkjær Nielsen, Søren M. Karst, Vincent Cattoir, Reto Lienhard, Andrea J. Grisold, Anne Buchhave Olsen, Mark Reinhard, Karen Marie Søby, Jens Jørgen Christensen, Jørgen Prag, Trine R. Thomsen

**Affiliations:** a Center for Microbial Communities, Department of Biotechnology, Aalborg University, Aalborg, Denmark; b Department of Clinical Microbiology, Viborg Regional Hospital, Viborg, Denmark; c CHU de Caen, Department of Microbiology, Caen, France; d ADMED, La Chaux-de-Fonds, Switzerland; e Institute of Hygiene, Microbiology and Environmental Medicine, Medical University of Graz, Graz, Austria; f Department of Urology, Viborg Regional Hospital, Viborg, Denmark; g Department of Internal Medicine, Viborg Regional Hospital, Viborg, Denmark; h Department of Clinical Microbiology, Slagelse Hospital, Slagelse, Denmark; i Section for Medical Biotechnology, Danish Technological Institute, Aarhus, Denmark

## Abstract

Complete genome sequencing of the emerging uropathogen *Actinobaculum schaalii* indicates that an important mechanism of its virulence is attachment pili, which allow the organism to adhere to the surface of animal cells, greatly enhancing the ability of this organism to colonize the urinary tract.

## GENOME ANNOUNCEMENT

*Actinobaculum schaalii* is an emerging commensal uropathogen in the genus *Actinobaculum* (phylum *Actinobacteria*, class *Actinobacteria*, order *Actinobacteridae*, family *Actinomycetales*) closely related to the genera *Actinomyces* and *Arcanobacterium. A. schaalii* is a facultative Gram-positive coccoid rod requiring CO_2_ for optimal growth. *A. schaalii* is highly susceptible to β-lactams, but it is resistant to trimethoprim, ciprofloxacin, and co-trimoxazole, the first-choice oral antibiotics for urinary tract infections (1–7).

Since its genome has not been sequenced yet, it is unknown how *A. schaalii* exercises its pathogenicity, which makes it interesting to study virulence genes of importance for establishing infection in the urinary tract.

*Actinobaculum schaalii* strain CCUG 27420 was incubated on Columbia agar with 5% sheep blood (Becton Dickinson, Heidelberg, Germany) in an atmosphere of 5% CO_2_ at 35°C for 2 days. Genomic DNA from the organism was isolated using the Ultra-Clean Microbial DNA Isolation Kit (MO BIO Laboratories, Inc., USA) followed by Agencourt AMPure XP bead purification (Beckman Coulter, Inc., Denmark) according to the manufacturer’s instructions. From 1 µg of purified genomic DNA, a sequencing-ready library was constructed using the Nextera MatePair sample preparation kit (Illumina, United States) according to the manufacturer’s instructions. The library was paired-end sequenced (2 × 300 bp) on an Illumina MiSeq instrument using a MiSeq Reagent kit (version 2). The reads were assembled *de novo* using the build-in tool of the CLC Genomics Workbench version 7.0 (CLC bio, USA). Manual scaffolding of contigs was carried out based on mate-pair information. Cytoscape version 2.8.3 (8) was used for visualization and manual inspection of the assemblies as described by Albertsen et al. (9). Gaps were closed by manual read mapping in CLC Genomics version 7.0. The average coverage of the assembly was 113×. Annotation was done using the NCBI Prokaryotic Genome Automatic Annotation Pipeline (PGAAP) (10), the Rapid Annotation using Subsystem Technology (RAST) server (11), and Magnifying Genomes (MaGe) software (12).

The complete *A. schaalii* CCUG 27420 genome is 2.16 Mbp with 1,444 predicted coding sequences. Of these, 545 were characterized as hypothetical proteins due to incomplete information about the genes in the databases. The coding sequences counted genes coding for proteins involved in regulation and cell signaling, cell wall and capsule formation, and membrane transport.

Genomic investigation of *A. schaalii* CCUG 27420 gave insight to the mechanisms responsible for virulence and resistance against some types of antimicrobial agents. The findings showed that the genome of *A. schaalii* codes for fimbrial genes, which are responsible for the production of attachment pili. The genomic investigation also showed that *A. schaalii* potentially is resistant to several antimicrobial agents.

Genomic investigation of pathogenic bacteria is a major key for a better understanding of virulence and resistance traits of these organisms, resulting in prevention and improved treatment of infections in the future.

### Nucleotide sequence accession number.

The whole-genome sequence of *A. schaalii* CCUG 27420 was deposited at GenBank under the accession number CP008802.
